# NRP1 promotes prostate cancer progression via modulating EGFR-dependent AKT pathway activation

**DOI:** 10.1038/s41419-023-05696-1

**Published:** 2023-02-25

**Authors:** Peng Zhang, Liang Chen, Fenfang Zhou, Zhiwen He, Gang Wang, Yongwen Luo

**Affiliations:** 1 Department of Urology, Zhongnan Hospital of Wuhan University, Wuhan, China; 2 Department of Biological Repositories, Zhongnan Hospital of Wuhan University, Wuhan, China; 3 Human Genetics Resource Preservation Center of Hubei Province, Wuhan, China; 4 Laboratory of Precision Medicine, Zhongnan Hospital of Wuhan University, Wuhan, China; 5 Medical Research Institute, Wuhan University, Wuhan, China

**Keywords:** Prostate cancer, Cell migration

## Abstract

Prostate cancer (PCa) is the most common malignant tumor with a high global incidence in males. The mechanism underlying PCa progression is still not clear. This study observed that NRP1 was highly expressed in PCa and associated with poor prognosis in PCa patients. Functionally, NRP1 depletion attenuated the proliferation and migration ability of PCa cells in vitro and in vivo, while NRP1 overexpression promoted PCa cell proliferation and migration. Moreover, it was observed that NRP1 depletion induced G1 phase arrest in PCa cells. Mechanistically, HIF1α is bound to the specific promoter region of NRP1, thereby regulating its transcriptional activation. Subsequently, NRP1 interacted with EGFR, leading to EGFR phosphorylation. This study also provided evidence that the b1/b2 domain of NRP1 was responsible for the interaction with the extracellular domain of EGFR. Moreover, EGFR mediated NRP1-induced activation of the AKT signaling pathway, which promoted the malignant progression of PCa. In addition, the administration of NRP1 inhibitor EG01377 significantly inactivated the EGFR/AKT signaling axis, thereby suppressing PCa progression. In conclusion, the findings from this study highlighted the molecular mechanism underlying NRP1 expression in PCa and provide a potential predictor and therapeutic target for clinical prognosis and treatment of PCa.

## Introduction

Prostate cancer (PCa) is the most common malignant tumor with a high incidence in males. According to the 2020 global cancer statistics, PCa is the second most frequent cancer diagnosis made and the fifth leading cause of cancer death in men, with an estimated 1.4 million new cases and 375,000 deaths occurring annually [1]. To date, androgen-deprivation therapy (ADT) is a critical treatment option for locally advanced and metastatic PCa, but a majority of patients ultimately acquire resistance to ADT and develop lethal castration-resistant prostate cancer (CRPC) [2]. It is important to note that there is still no effective treatment strategy for patients with CRPC as the mechanism of PCa progression is obscure, and this has become a bottleneck restricting the treatment of PCa. Therefore, it is notably urgent to study and determine key molecules in the mechanism of PCa progression, detect potential drug treatment targets, and improve the survival prognosis of patients.

Neuropilin 1 (NRP1) is a single-pass transmembrane glycoprotein, consisting of extracellular, transmembrane, and intracellular domains [3]. The extracellular domain mediates interaction with other proteins and is mainly composed of three structural motifs: the CUB domain (a1/a2 domain), the coagulation factors V/VIII domain (CF or b1/b2 domain), and the MAM domain (c domain) [4, 5]. Previous studies have indicated that NRP1 as a co-receptor protein can bind with several extracellular ligands, including semaphorin (SEMA), placental growth factor-2 (PLGF-2), hepatocyte growth factor (HGF), platelet-derived growth factor (PDGF), transforming growth factor β (TGF-β), etc., thereby activating several downstream oncogenic signaling pathways [6–8]. It has been reported that NRP1 affects coronavirus disease 2019 (COVID-19) infectivity, immune responses, cardiovascular and nervous system development, and angiogenesis [9–12]. Recently, increasing evidence suggested that NRP1 plays a critical role in various cancers [10, 13, 14]. For example, NRP1 is overexpressed in claudin-low breast cancer and promotes tumor progression via the RAS/MAPK pathway [15]. NRP1 can interact with CMTM6 and promote the progression of oral squamous cell carcinoma [16]. Moreover, NRP1 is not only important for the intrinsic stability of Treg cells in the tumor microenvironment but also significantly inhibits the anti-tumor function of CD8^+^ T cells. Therefore, NRP1 can be a checkpoint target with significant implications for cancer immunology and immunotherapy [17, 18].

Few researchers have previously revealed the oncogenic role of NRP1 in PCa. A retrospective case-control study reported that NRP1 endothelial expressions predicted distant relapse after radical prostatectomy in clinically localized PCa [19]. Another study showed NRP1 expression to be an independent prognostic biomarker of metastatic progression and patient mortality in PCa. Moreover, that study also reported that NRP1 promoted adaptive response to androgen-targeted therapies (ATTs) and progression to CRPC [20]. Moreover, NRP1-targeted gold nanoparticles were found to enhance the therapeutic efficacy of the platinum drugs in PCa treatment [21]. However, the role and mechanism of NRP1 in PCa are still unclear and need further research.

In this study, NRP1 was identified as a novel marker for PCa progression through transcriptome profiling, and the overexpression of NRP1 was correlated with unfavorable prognosis in PCa patients. The b1/b2 domain of NRP1 interacts with the extracellular domain of EGFR in a mechanistic manner to induce the activation of EGFR, which in turn, activates the AKT pathway, thereby promoting the proliferation and migration of PCa cells. Moreover, HIF1α is involved in the transcriptional regulation of NRP1. The findings from this study highlighted that the inhibition of the NRP1/EGFR axis can be a potential therapeutic approach for PCa.

## Materials and methods

### Gene expression profiles

The RNA-Seq data of the TCGA-PRAD and HRA000099 cohorts were downloaded from the TCGA database and The Genome Sequence Archive for Human, respectively. The RNA sequence analysis (siControl and siNRP1) was performed at Beijing Genomic Institute (BGI). The “EdgSeq” R package was used for the normalization of mRNA-Seq data. The microarray datasets of GSE10645 and GSE70769 were downloaded from the GEO database (https://www.ncbi.nlm.nih.gov/geo/) on the Illumina platform, and the “limma” R package was used to process the gene microarray data. Gene set enrichment analysis (GSEA), Gene set variation analysis (GSVA) and KEGG pathway analysis were carried out to evaluate the pathways enriched in the NRP1 high- and low-expression subgroups. The msigdbr, enrichplot, clusterProfiler, and ggplot2 packages in R were used for analysis and visualization.

### Tissue microarray and human PCa tissue samples

Tissue microarray (TMA) containing samples from paired 80 tumor and paracancerous tissue specimens were purchased from Shanghai Weiao Biotechnology Co., Ltd (ZL-PRC1601; Shanghai, China) and colored according to the technical manual containing the TMA pathologic information, which is available from the website (http://www.biotechwell.com/index.php?m=home&c=View&a=index&aid=4610). During the experiments, five tissue sections were missing from the tissue microarray. Therefore, 75 tissue specimens were included for the statistical analysis. Ten PCa tissue specimens were generated from patients who underwent surgery at Zhongnan Hospital of Wuhan University. These tissues were formalin-fixed and paraffin-embedded for subsequent experiments. The pathological diagnosis of PCa tissue specimens was independently validated by two pathologists. This study was approved by the Ethics Committee of Zhongnan Hospital of Wuhan University (approval number: 20200507), and the specimens met the defined standards of patient information and specimens. All the patients had signed informed consent before the study.

### Cell culture and reagents

All the cell lines were purchased from the Stem Cell Bank, Chinese Academy of Sciences in Shanghai, China, and verified by the China Centre for Type Culture Collection in Wuhan, China. PCa cell lines (PC-3, DU-145, LNCap, and 22RV1) were cultured in Gibco RPMI-1640 medium (Thermo Fisher Scientific, USA). HEK293T cell line was cultured in Gibco DMEM medium (Thermo Fisher Scientific, USA). All the cell cultures were supplemented with 10% fetal bovine serum (FBS) (Gibco; Thermo Fisher Scientific, USA) and cultured at 37 °C in a humidified incubator containing 5% CO_2_. Gefitinib (S1025, Selleck), Cycloheximide (S7418, Selleck), Dactinomycin (S8964, Selleck), and EG01377 (HY-112151, MCE) were purchased from commercial sources indicated.

### Plasmids, RNA inference, retroviral infection, and cell transfection

To construct expression plasmids encoding the human full-length NRP1 (NM_003873.7), HIF1α (NM_001243084.2), and EGFR (NM_001346897.2), the full lengths of NRP1, HIF1α, and EGFR protein-coding sequences were commercially synthesized and subcloned into pcDNA3.1 vector (Obio Technology). Then, full-length NRP1 and the corresponding truncated plasmids were subcloned into a pcDNA3.0-HA vector, whereas full-length EGFR and the corresponding truncated plasmids were subcloned into a pcDNA3.0-Flag vector. The small interfering RNAs targeting NRP1 (sense sequence of NRP1-siRNA1 was 5’-GCUCUGGAAUGUUGGGUAUTT-3’, antisense sequence of NRP1-siRNA1 was 5’- AUACCCAACAUUCCAGAGCTT-3’, sense sequence of NRP1-siRNA2 was 5’-CCACAAGUCUCUGAAACUUTT-3’, antisense sequence of NRP1-siRNA2 was 5’-AAGUUUCAGAGACUUGUGGTT-3’, negative control sense sequence was 5′-UUCUCCGAACGUGUCACGUTT-3′, and antisense sequence was 5’-UAUCGUCUGUGCAAUUAGCTT-3’) were obtained from Genomeditech (Shanghai, China). The lentiviral vector containing short hairpin RNA for NRP1 was also constructed and identified by Genomeditech (Shanghai, China). Lipofectamine 3000 (Invitrogen, Carlsbad, CA, USA) was used for cell transfection in accordance with the manufacturer’s instructions.

### RNA extraction, reverse transcription, and quantitative real-time PCR (qRT-PCR)

Total RNA extraction was performed using RNeasy plus mini kits (Qiagen, Germany) following the manufacturer’s instructions. Subsequently, the quality of the extracted RNA was determined using the NanoDrop instrument (Implen, Germany). Then, complementary DNA was synthesized using the ReverTra Ace qPCR RT Kit (Toyobo, Japan). Finally, the qRT-PCR was performed on the QuantStudio 6 Flex System (Life Technologies). The specific primer sequences are shown in Supplementary Table S1. GAPDH was used as the internal control, and the expression of the targeted genes was normalized to GAPDH.

### Cell proliferation assay

The clone formation assay and methyl thiazolyl tetrazolium (MTT) assay were used to detect cell proliferation and viability. For the colony-formation assay, the cells were seeded into six-well plates at 800 cells/well in the desired medium. After 14 days, cells were washed with PBS, fixed with 4% paraformaldehyde (PFA), and stained with 0.5% crystal violet. For the MTT assay, 3 × 10^3^ cells were seeded into 96-well plates (6 replicate wells per plate) for growth. Then, the 96-well plates were taken out of the incubator every 24 h for five consecutive days, and 20 μL MTT reagent (5 mg/mL, Sigma-Aldrich) was added to each well. The plates were then placed again inside the incubator for 4 h, then 150 μL DMSO was added to each well. Finally, the absorbance of each well at 570 nm was tested using a microplate reader (Molecular Devices, USA) to assess cell viability.

### Flow cytometry analysis

The transfected PCa cells were sequentially digested and centrifuged. For cell cycle analysis, 1 × DNA staining solution (Multisciences, China) supplemented with propidium iodide was used to treat the cells for 30 min at room temperature. FITC Annexin V Apoptosis Detection Kit I (BD Biosciences, USA) was used to stain cells for analysis of cell apoptosis. Then, the stained cells were incubated for 30 min at room temperature. The flow cytometry (cat. no. FC500; Beckman Coulter, USA) was applied in the analysis of cell cycle and cell apoptosis.

### Cell migration assay

Wound-healing assay and transwell assay were applied for detecting cell migration ability. For the transwell assay, 1 × 10^4^ cells in 200 μL serum-free medium were seeded into transwell inserts (Corning, USA) and placed in 24-well plates with 800 μL medium containing 10% FBS. After 24 h incubation, the cells were fixed with 4% PFA and stained with crystal violet. Finally, the samples were observed for recording images of cell migration. For the wound-healing assay, the transfected PCa cells were cultured in six-well plates to 80% density. Then, the cells were vertically scratched using a 200 mL pipette, and the scratched cells were then washed with PBS. 2 mL of culture medium was then added for further cultivating the cells for 12 h. Finally, the cells were observed to calculate the average wound gap between wound edges.

### Immunohistochemical (IHC) analysis

IHC assay was performed according to the following protocol. Briefly, tissue specimens were fixed with 4% formalin and embedded in paraffin. Further, thin sections of tissue embedded in paraffin were cut, dewaxed, and rehydrated. The sections were then blocked in 10% goat serum at room temperature for 10 min and incubated overnight at 4 °C in primary antibodies. Then the sections were incubated in the appropriate secondary antibody and processed with streptavidin-peroxidase before counterstaining with hematoxylin. Specific primary antibodies against NRP1 (Abcom, cat# ab81321), EGFR (CST, cat# 4264), p-EGFR (CST, cat# 2234), p-AKT (CST, cat# 4060 L), p-mTOR (Abcam, cat# ab109268), and KI67 (Abcom, cat# ab15580) were used for IHC. The images were scanned and analyzed using a molecular microscope (Olympus BX53). TMA and IHC analyses were generally performed as described previously [22]. The IHC staining results were quantified by multiplying the staining intensity (strong = 3, moderate = 2, weak = 1, and negative = 0) with the percentage of immunoreactive cells (81–100% = 4, 51–80% = 3, 11–50% = 2, 1–10% = 1, and 0% = 0). The final scores were defined as strongly positive (3 + ; 9–12), moderately positive (2 + ; 6–8), weakly positive (1 + ; 2–4), and negative (0; 0–1).

### Immunofluorescence assay

PC-3 and DU-145 cells were washed with PBS followed by fixation with 4% PFA for 20 min at room temperature. The cells were again washed thrice with PBS and then incubated overnight at 4 °C in desired antibodies. After washing thrice in PBS again, the cells were incubated in fluorescein-labeled secondary antibodies for 40 min at 37 °C. After washing thrice again with PBS, cells were incubated in DAPI (D8417, Sigma-Aldrich, Germany) at room temperature for 30 min and mounted in the mounting medium. The slides were then analyzed using a laser scanning confocal microscope (LSM880, Zeiss, Germany).

### Immunoblot assay

The total cellular protein was isolated using RIPA lysis buffer (Beyotime, China) supplemented with protease inhibitors (Sigma-Aldrich, USA). Equal amounts of total proteins were loaded on SDS-PAGE gels for separation of proteins according to their molecular weights and then transferred onto PVDF membranes (Merck Millipore, Billerica, MA, USA). The membranes were blocked with 5% non-fat milk in Tris-buffered saline with Tween-20 and then incubated with primary antibodies overnight at 4 °C, followed by incubation with corresponding secondary antibodies at room temperature for 2 h. The protein bands were visualized using enhanced chemiluminescence. Immune response bands were exposed on a Bio-Rad ChemiDoe XRS + Imaging System (Bio-Rad, USA). The primary antibodies used were NRP1 (Abcom, cat# ab81321), EGFR (CST, cat# 54359), p-EGFR (CST, cat# 2234), CDK2 (Abcam, cat# ab32147), CDK4 (CST, cat# 12709), CDK6 (CST, cat# 13331), CyclinD1 (CST, cat# 2922), E-cadherin (CST, cat# 3195S), N-cadherin (CST, cat# 13116S), AKT (CST, cat# 4691L), p-AKT (CST, cat# 4060L), GSK3β (CST, cat# 12456S), p-GSK3β (CST, cat# 5558S), mTOR (Abcam, cat# ab32028), p-mTOR (Abcam, cat# ab109268), FLAG (F1804, Sigma), HA (TA180128, OriGene), and GAPDH (Santa Cruz, cat# sc-365062).

### Co-immunoprecipitation

Co-immunoprecipitation (Co-IP) was performed as described in a previous study [23]. The cells were lysed in lysis buffer (Aspen Biological) and approximately 200 μg of total cellular proteins were incubated overnight with target antibody at 4 °C followed by the addition of 20 μL of protein A agarose beads (#1614813, BIO-RAD). Rabbit control IgG (AC005, Abclonal, Wuhan, China) was used in the reaction as the negative control. The precipitates were washed with lysis buffer and boiled in SDS sample buffer. The supernatant was then subjected to immunoblotting.

### Dual-luciferase assay

The possible binding sites of HIF1α on the promoter region of the human NRP1 gene were predicted using JASPAR matrix models. Based on the predicted sequences of the binding sites, full-length fragments containing the putative human NRP1 promoter region or a negative control region (Obio Technology) were cloned into the pGL4.1-basic plasmid. The pcDNA3.1-HIF1α overexpressing plasmid was also constructed (OBIO Technology). The constructed vectors were verified by sequencing. The PC-3 and DU-145 cells were co-transfected with specific pGL4.1-basic NRP1 promoter plasmids and Renilla luciferase reporter plasmid (pRL-TK) or pcDNA3.1-HIF1α expression plasmid. The luciferase activities were measured 48 h after transfection using the Dual-Luciferase Reporter Assay System (RG027, Beyotime Biotechnology, Shanghai, China). The ratio of firefly luciferase activity/Renilla luciferase activity was also calculated.

### In vivo tumorigenesis and metastasis assay

Animal experiments were performed following the National Institutes of Health Animal Use Guidelines. All the experimental protocols were approved by the Institutional Animal Care and Use Committee of Wuhan University. The 3-week-old male BALB/c-nu mice (Charles River Laboratories, Beijing, China) were raised under a specific pathogen-free (SPF) environment. After 1-week adaption, the mice were randomly divided into 2 groups. For the xenograft mice model, PC-3 cells (5 × 10^6^ in 100 µL serum-free medium) infected with LV-control (NC group) or LV-shRNA (sh-NRP1 group) lentivirus vectors were injected subcutaneously in the dorsal flank of each mouse. The tumor length and width were measured by a caliper every 5 days using the equation, i.e., tumor size (mm^3^) = (length × width^2^)/2. The xenograft growth was monitored for 6 weeks after injection. All the mice were euthanized to obtain fresh tumors and the tumor weights were recorded. Further, the xenografts were analyzed for the desired antibodies by hematoxylin and IHC staining. For the pulmonary metastasis model, the above cells (1 × 10^6^ cells in 100 µL serum-free medium) were injected into the tail vein of each mouse. After growth for another 8 weeks, the fluorescence intensity of tumors was measured using the Living Image software (Caliper Life Sciences). H&E staining of the lung tissues obtained from mice was used to evaluate lung morphology. The investigator was blinded to the treatment group to which the animals were allocated during the experiment.

### Molecular docking

The three-dimensional structures of NRP1 and EGFR were drawn in Chem3D Ultra 14.0 software (CambridgeSoft, USA). The crystal structures of NRP1 (PDB ID: 2QQI) and EGFR (PDB ID:2GS2) proteins were obtained from the RCSB PDB database (https://www1.rcsb.org/). The central site of the docking box was determined using AutoDock Tools (version 1.5.6), and AutoDock Vina was used for the virtual molecular docking. Then, the docking image of the best binding pose was generated by PyMOL (version 1.7.2.1).

### Statistical analysis

R software (version: 3.5.2) and GraphPad Prism version 6.0 (GraphPad Software, USA) were used for statistical processing and plotting. Continuous data were presented as the mean ± SD. The differences in mean values between the two groups were compared using a two-tailed *t* test for normally distributed data or the Mann–Whitney *U* test for non-normally distributed data. The means of multiple groups were compared using one-way ANOVA. The categorical data were expressed as a component ratio or rate, while a chi-square test or continuity correction of chi-square test was used for comparison of the constituent ratios between NRP1 high-expression and low-expression groups. Spearman’s test was used to evaluate the correlations between variables. Kaplan–Meier method was used for survival analysis. Univariate and multivariate Cox proportional hazard models were used to identify independent prognostic factors of PCa patients. *P* < 0.05 was considered statistically significant.

## Results

### NRP1 is highly expressed in PCa tissues and could be a prognostic biomarker for PCa patients

**Fig. 1:**
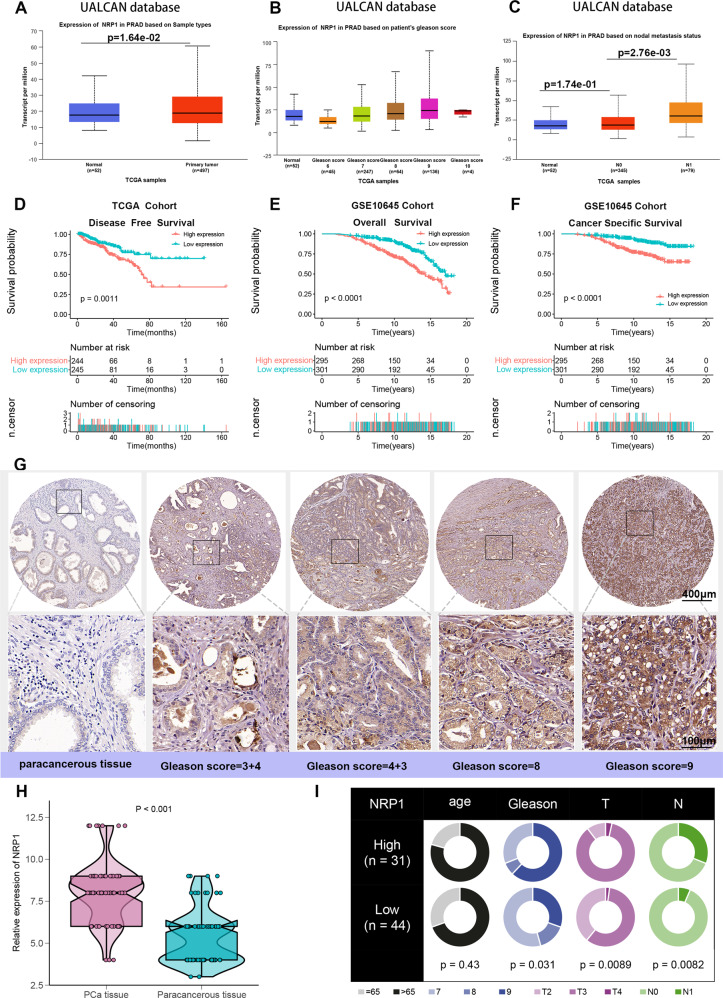
NRP1 is overexpressed in PCa tissues and associated with unfavorable prognosis of PCa patients. **A** Differential expression of NRP1 gene in PCa and adjacent tissues. **B** NRP1 expression is associated with higher Gleason scores in PCa. **C** NRP1 expression is associated with lymphatic metastasis in PCa. **A**–**C** Complete data are obtained from the UALCAN database. **D**–**F** Kaplan–Meier survival analysis of PCa patients from the TCGA and GSE10645 datasets. **G** IHC analysis of NRP1 expression in 75 PCa samples. Representative images are shown, with the scale bar of 400 μm and 100 μm, respectively. **H** Differential expression of NRP1 protein in 75 PCa tissues and paired paracancerous tissues (statistical significance assessed using a Mann–Whitney *U* test). **I** Comparison of age, Gleason score, T stage, and N stage between high NRP1 expression group and low NRP1 expression group (grouping based on the median of IHC staining score, and statistical significance assessed using the chi-square test).

The PCa dataset was analyzed in the UALCAN database to evaluate NRP1 expression in PCa clinical samples. The results revealed that NRP1 was significantly overexpressed in PCa tissues when compared with normal prostate tissues (Fig. 1A, *P* < 0.05). Moreover, NRP1 expression gradually increased with the increase in Gleason score, and NRP1 expression was significantly higher in PCa patients with lymph node metastasis when compared with those without lymph node metastasis (Fig. 1B, C, *P* = 2.76e-03). Further, the correlation between NRP1 and clinicopathological characteristics in the TCGA-PRAD cohort was evaluated. As shown in Table 1, NRP1 expression is significantly correlated with PSA levels, Gleason score, advanced T stage, and lymphatic metastasis. Kaplan–Meier analysis indicated that higher levels of NRP1 were associated with an unfavorable disease-free survival (DFS) (*P* = 0.0011; Fig. 1D) in the TCGA-PRAD cohort. The GSE10645 PCa cohort results demonstrated that PCa patients in the high NRP1 expression group indicated shorter overall survival (OS) rate and cancer-specific survival (CSS) rate than those in the low NRP1 expression group (*P* < 0.0001; *P* < 0.0001; Fig. 1E, F). Furthermore, multivariate Cox proportional hazard analysis revealed that PCa patients with NRP1 high expression indicated a worse DFS than those with NRP1 low expression (HR = 1.45, *P* = 0.037; Table 2). The above results were validated by assessing the NRP1 protein expression in PCa using tissue microarray (TMA). This result also indicated that NRP1 protein expression in PCa tumor tissues was higher than those in paracancerous tissues (Fig. 1G, H). By choosing the median IHC score (8.0) as the cut-off value, 75 patients were divided into the low NRP1 group (*n* = 44) and the high NRP1 group (*n* = 31). The chi-square analysis indicated that high NRP1 expression was positively associated with higher Gleason score, advanced stage, and lymphatic metastasis (Fig. 1I). Therefore, these results demonstrated that NRP1 may promote PCa progression and may be a potential prognosis factor for PCa patients.

**Table 1:** Association between the expression of NRP1 and clinicopathological characteristics.

Variable	Total patients	NRP1 expression		*P* value
	No. (%)	Low	High	
Age, mean ± SD, (years)	61.0 ± 12	61.1 ± 12.5	60.9 ± 10.5	0.677
Age				0.284
<=65	349 (71.4%)	169 (69.0%)	180 (73.8%)	
>65	140 (28.6%)	76 (31.0%)	64 (26.2%)	
PSA				0.034
<=10	417 (96.5%)	214 (98.6%)	203 (94.4%)	
>10	15 (3.5%)	3 (1.4%)	12 (5.6%)	
Gleason score				0.000259
<=8	352 (72.0%)	195 (79.6%)	157 (64.3%)	
>8	137 (28.0%)	50 (20.4%)	87 (35.7%)	
T stage				6.07e-07
T2	185 (37.5%)	120 (49.8%)	65 (26.9%)	
T3	298 (60.4%)	119 (49.4%)	169 (69.8%)	
T4	10 (2.1%)	2 (0.8%)	8 (3.3%)	
Lymphatic metastasis				0.00029
Negative	340 (81.5%)	173 (89.2%)	167 (74.9%)	
Positive	77 (18.5%)	21 (10.8%)	56 (25.1%)	
Biochemical recurrence				0.177
No	365 (86.7%)	189 (89.2%)	176 (84.2%)	
Yes	56 (13.3%)	23 (10.8%)	33 (15.8%)

**Table 2:** Univariate and multivariate analyses of risk factors for disease-free survival of prostate cancer.

Variable	Univariate analysis HR (95% CI)	*P* value	Multivariate analysis HR (95% CI)	*P* value
NRP1 (high/low)	1.65 (1.21–2.23)	0.001	1.45 (1.02–2.06)	0.037
Gleason score (>8/<=8)	2.83 (2.04–3.92)	<0.001	1.86 (1.21–2.85)	0.0046
Age (>65/<=65)	1.12 (0.81–1.54)	0.51	NA	NA
PSA (<=10/>10)	2.64 (1.39–5.03)	0.003	0.89 (0.43–1.88)	0.78
Stage (T4/T3/T2)	2.62 (1.74–3.95)	<0.001	1.07 (0.59–1.95)	0.82
Lymphatic metastasis (yes/no)	1.49 (1.06–2.11)	0.02	0.90 (0.61–1.32)	0.58
Surgical margin (positive/negative)	1.78 (1.33–2.39)	<0.001	1.60 (1.12–2.29)	0.01
Biochemical recurrence (yes/no)	4.56 (3.37–6.15)	<0.001	2.96 (2.07–4.24)	<0.001

### HIF1α binds the NRP1 promoter and increases its expression under hypoxic conditions

**Fig. 2:**
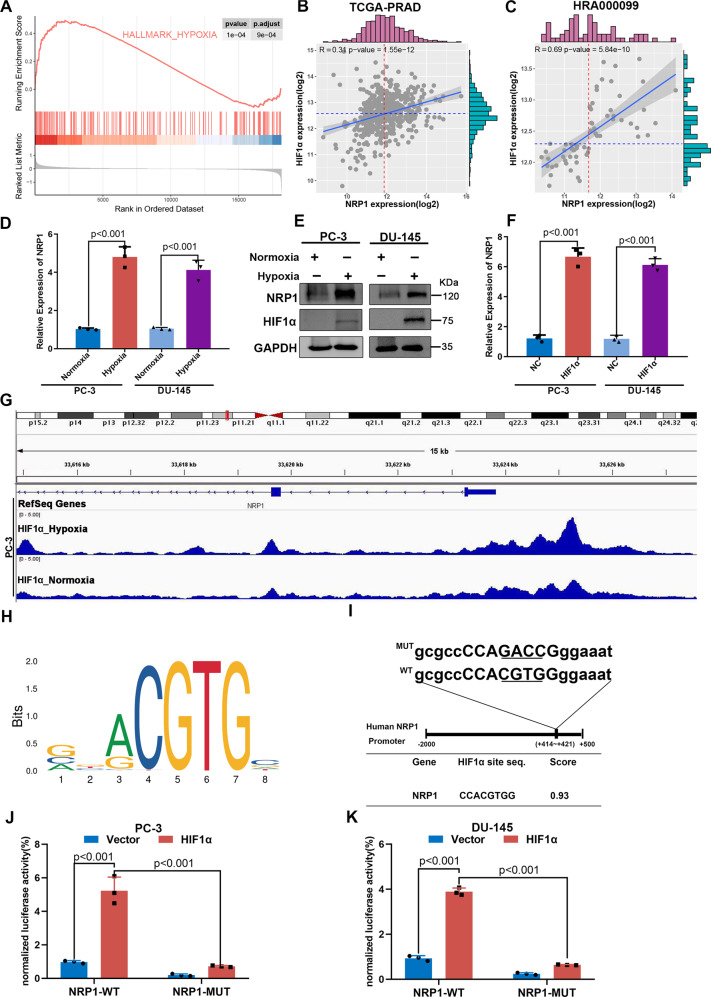
HIF1α increases the expression of the NRP1 gene through transcriptional activation in PCa cells. **A** GSEA analysis of the GSE70769 dataset indicated NRP1 is significantly associated with the hypoxia pathway. **B**, **C** HIF1α expression is correlated with NRP1 expression based on the TCGA-PRAD and HRA000099 datasets. **D** Expression of NRP1 mRNA level increases under hypoxic conditions. **E** Significant increase in protein levels of HIF1α and NRP1 in PCa cells under hypoxic conditions. **F** HIF1α overexpression promotes NRP1 mRNA expression. **G** GSE106305 ChIP dataset indicates HIF1α binding to the NRP1 promoter region shows a significant peak. **H** Schematic of canonical HIF1α motif binding sites provided by the JASPAR database. **I** Diagram represents the predicted HIF1α binding site on the NRP1 promoter, as well as wild-type/mutant NRP1 promoter plasmids. **J**, **K** Luciferase assay indicates that HIF1α significantly increases NRP1-WT promoter-driven luciferase activity, while the mutation of the binding site of HIF1α to NRP1 leads to significant attenuation of the luciferase activity in PC-3 and DU-145 cells, respectively. Values represent the mean ± SD of three independent experiments. Statistical significance was assessed using two-tailed *t* tests.

Hypoxia plays a critical role in the occurrence and progression of PCa [24–26]. GSEA results indicated that NRP1 was significantly associated with the hypoxia pathway in the GSE70769 dataset (Fig. 2A). HIF1α is a transcription factor associated with hypoxic stress and regulates several oncogenes to maintain and promote blood vessel formation and tumor growth in PCa. The correlation between NRP1 and HIF1α was analyzed based on the TCGA-PRAD and HRA000099 cohorts, indicating that the expression of NRP1 was significantly positively correlated with HIF1α (Fig. 2B, C). Further, it was determined whether NRP1 expression could be induced by hypoxia in PCa cells. DU-145 and PC-3 cells were grown in 1% oxygen under hypoxic conditions for 24 h, which led to enhanced NRP1 and HIF1α expression (Fig. 2E). Further, HIF1α was overexpressed using HIF1α plasmids, and NRP1 expression was found to increase with HIF1α (Fig. 2F). GSE106305 ChIP dataset results indicated significant peaks of HIF1α binding to NRP1 promoter region in PC-3 cells (Fig. 2G). Further, the JASPAR database (https://jaspar.genereg.net/) was used to predict the binding site of HIF1α on the NRP1 promoter region. Based on the canonical binding motifs of HIF1α (Fig. 2H), high-scoring motifs were observed between +414 and +421 upstream of the transcription start site. Then, the NRP1 wild-type (WT) plasmid and mutant (MUT) plasmid with HIF1α bound to the NRP1 promoter were constructed (Fig. 2I). The luciferase assay indicated that HIF1α expression significantly enhanced NRP1-WT promoter-driven luciferase activity, while the luciferase activity was significantly attenuated when the binding site of HIF1α to NRP1 was mutated (Fig. 2J, K). Overall, the findings from this study indicated that HIF1α transcriptionally activated NRP1 by binding to the NRP1 promoter.

### NRP1 promotes the proliferation and migration capacity of PCa cells

**Fig. 3:**
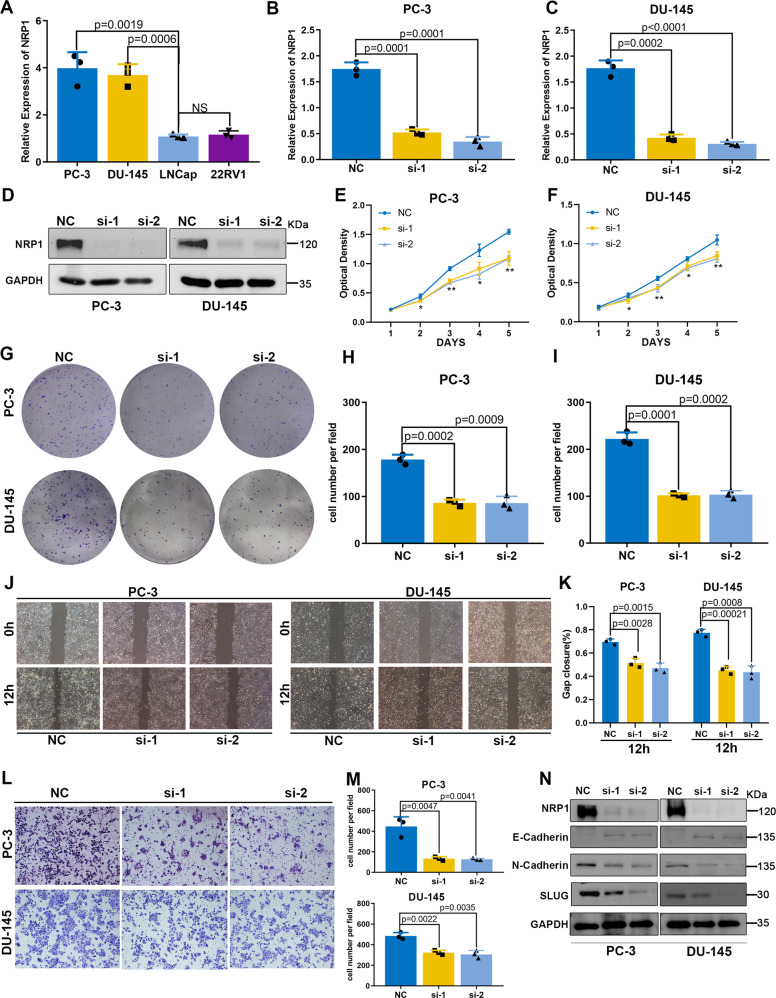
NRP1 depletion attenuates PCa cell proliferation and migration in vitro. **A** mRNA expression level of NRP1 detected in four PCa cell lines (PC-3, DU-145, LNCap, 22RV1) by qRT-PCR. **B**, **C** qRT-PCR analysis evaluates the depletion efficiency of two NRP1-specific siRNA in PC-3 and DU-145 cells, and si-1 and si-2 represent favorable depletion efficiency. **D** Immunoblot analysis validates depletion efficiency of si-1 and si-2 in PC-3 and DU-145 cells. **E**, **F** MTT assay reveals that NRP1 depletion attenuates cell viability in PC-3 and DU-145 cells. **G**–**I** Clone formation assay and the statistical chart represent NRP1 depletion attenuating clone formation ability in PC-3 and DU-145 cells. **J**, **K** Wound-healing assay and the statistical chart show that NRP1 depletion inhibits cell migration in PC-3 and DU-145 cells. **L**, **M** Transwell assay and the statistical chart demonstrate that NRP1 depletion inhibits cell migration in PC-3 and DU-145 cells. **N** Immunoblot assay shows EMT-related proteins in PC-3 and DU-145 cells after NRP1 depletion. Statistical significance was assessed using two-tailed t tests. Error bars represent the standard deviations of three independent experiments. *P < 0.01, **P < 0.001.

Four PCa cell lines (PC-3, DU-145, LNCap, and 22RV1) were chosen to investigate the biological function of NRP1 (Fig. 3A). As NRP1 was relatively highly expressed in PC-3 and DU-145 cell lines, NRP1 gene was depleted in the PC-3 and DU-145 cells (Fig. 3B, D). The MTT results demonstrated that the depletion of NRP1 significantly attenuated cell proliferation ability (Fig. 3E, F), while the clone formation assay revealed that NRP1 depletion significantly decreased the clone formation ability (Fig. 3G, I). As the cell cycle and apoptosis are two important factors affecting cell proliferation and growth, flow cytometry was applied for detecting the effect of NRP1 on the PCa cell cycle and apoptosis. The results implied that the proportion of cells in the G1 phase increased significantly after NRP1 depletion in the PC-3 and DU-145 cells (Supplementary Fig. S1A, B), indicating that NRP1 depletion inhibited cell proliferation by inducing cell cycle arrest at the G1 phase. The apoptotic proportion of PCa cells slightly increased after NRP1 depletion; however, the statistically significant difference between the groups was not observed (Supplementary Fig. S1C, D). The expression levels of CDK2, CDK4, CDK6, and CCND1 were attenuated in NRP1-depleted cells when compared to the control cells (Supplementary Fig. S1E, F). Therefore, all the results suggested that NRP1 promoted cell proliferation by inducing cell cycle G1 transition in PCa cells. Further, the altered migration ability of both PC-3 and DU-145 cells was detected using the transwell assay and wound-healing assay. The depletion of NRP1 led to the attenuation in the migration ability of PCa cells (Fig. 3J–M). Epithelial–mesenchymal transition (EMT) is regarded as a pathological process leading to tumor progression, which enables the tumor cells to acquire infiltrating and metastasizing properties. EMT marker (E-cadherin, N-cadherin, and slug) expressions were analyzed by immunoblot assay. The results indicated E-cadherin upregulation and downregulation of both N-cadherin and slug after NRP1 depletion (Fig. 3N). Thus, NRP1 may promote the migration of PCa cells by regulating EMT. In this study, NRP1 was concomitantly overexpressed in LNCap and 22RV1 cells (Supplementary Fig. S2A, B). Contrarily, ectopic overexpression of NRP1 promoted PCa cell proliferation and migration (Supplementary Fig. S2C–I).

### Inhibition of NRP1 blocks tumor growth and metastasis in vivo

**Fig. 4:**
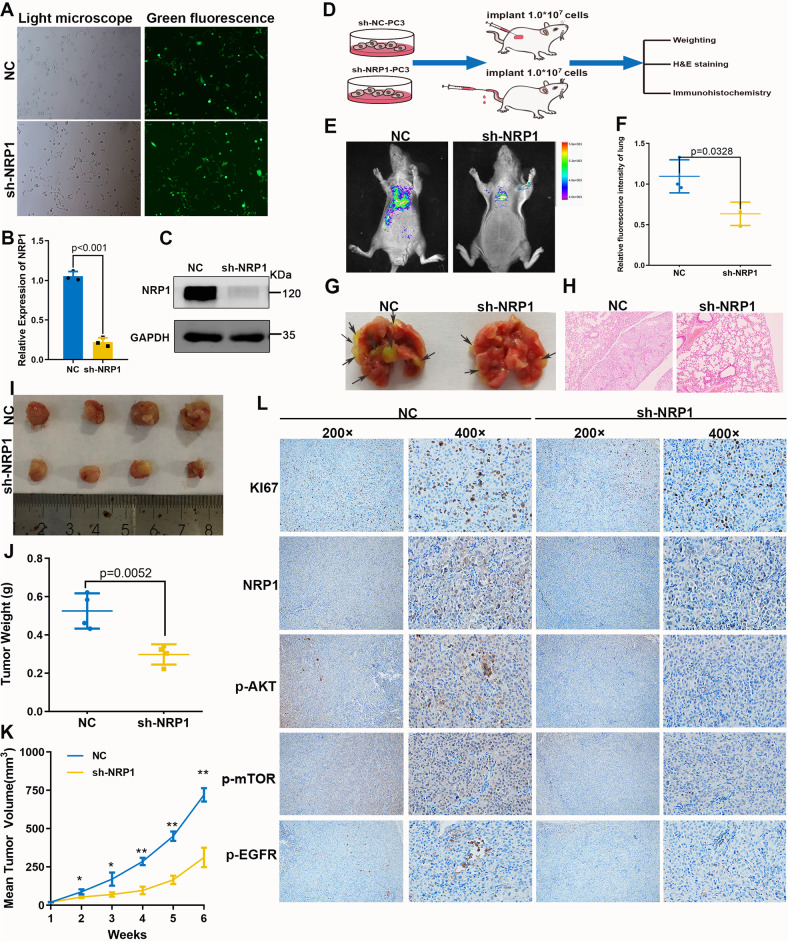
NRP1 promotes PCa cell proliferation and migration in vivo. **A** Green fluorescence of PC-3 stable cells. **B**, **C** qRT-PCR and immunoblot assay verify NRP1-depletion efficiency in PC-3 stable cells. **D** Xenograft mice models and pulmonary metastasis models were established by subcutaneously injecting LV-NC cells or LV-sh-NRP1 cells. Mice were monitored continuously for 5 weeks in xenograft mice models and 8 weeks in the pulmonary metastasis model. Then the mice were sacrificed and the tumors were dissected. **E** Fluorescence intensity of pulmonary metastasis is detected by imaging apparatus for small animals in vivo, **F** sh-NRP1 group (*n* = 3) significantly inhibited lung metastasis of PCa cells compared with that in the NC group (*n* = 3). **G** Lung tissue specimens show pulmonary metastatic nodules, where the arrows denote the tumors transferred to the lungs. **H** H&E staining of lung tissues (scale bar = 100 μm). Statistical analysis of tumor volume and weight in two groups (*n* = 4 in each group). **I** Tumor image of xenograft mice models. **J** NRP1 depletion significantly decreases the weight of the tumor xenograft. **K** NRP1 depletion significantly inhibits tumor growth. **L** IHC staining detects the expression of Ki67, NRP1, p-AKT, p-mTOR, and p-EGFR. Data are shown as the mean ± SD. Statistical significance was assessed using a two-tailed t test. *P < 0.01, **P < 0.001.

Further, the role of NRP1 in tumor growth and migration in the xenograft mouse model was investigated. Firstly, PC-3 cell lines with stable depletion of NRP1 by lentivirus-based shRNA were obtained (Fig. 4A), and the depletion efficiency of NRP1 was confirmed by qRT-PCR and immunoblot assay (Fig. 4B, C). Then, PC-3 LV-control cells and PC-3 LV-sh-NRP1 cells were separately injected into BALB/c-nu mice to construct a xenograft mice model and a pulmonary metastasis model (Fig. 4D). In the pulmonary metastasis model, sh-NRP1 significantly inhibited lung metastasis of PCa cells compared with NC group (Fig. 4E–H). In the xenograft mice model, the sh-NRP1 group had slower tumor growth compared with the NC group (Fig. 4I, J). Therefore, the average weights of tumors in the sh-NRP1 group were significantly lighter than those in the NC group (Fig. 4K). Moreover, the dissected tumors were assessed by H&E and IHC staining. The H&E staining demonstrated that the sh-NRP1 group had a lower degree of nucleus atypia, while the IHC staining revealed worse positive-staining of Ki67, p-EGFR, p-AKT, and p-mTOR in the sh-NRP1 group than in the NC group (Fig. 4G). Thus, these results indicated that the depletion of NRP1 inhibited PCa cell growth and migration in vivo.

### NRP1 interacts with the EGF receptor (EGFR) and modulates EGFR activation

**Fig. 5:**
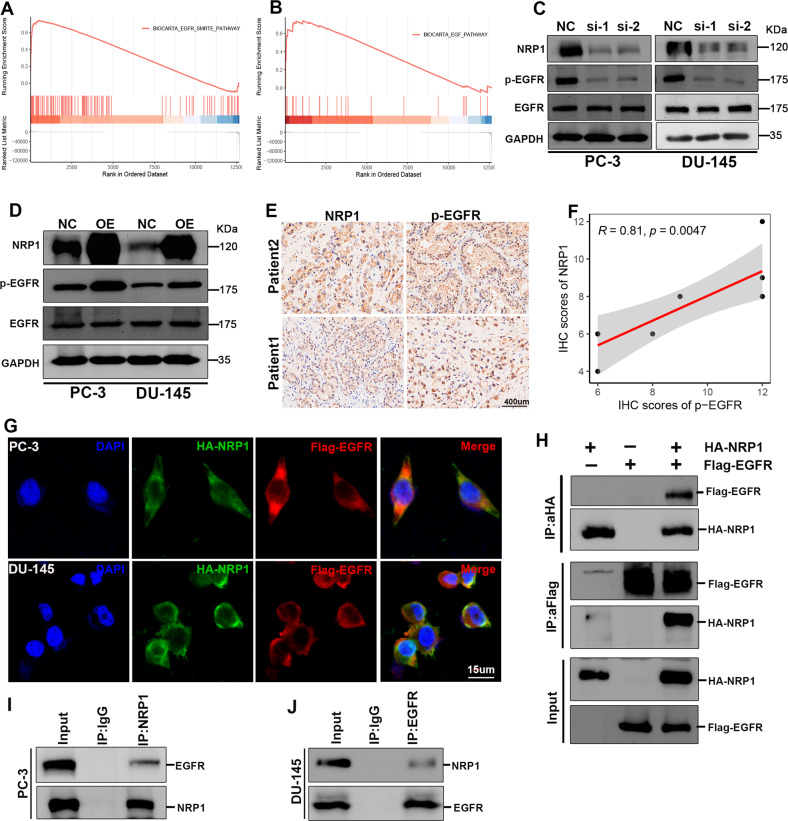
NRP1 interacts with EGFR and induces EGFR phosphorylation. **A**, **B** GSEA of TCGA-PRAD shows NRP1 is positively related to the EGFR pathway. **C**, **D** Immunoblot assay elucidates that NRP1 depletion attenuates EGFR phosphorylation level, while ectopic overexpression of NRP1 promotes EGFR phosphorylation level. **E**, **F** IHC assay and the correlation analysis represent that NRP1 protein is positively correlated with the expression of phosphorylated EGFR protein in PCa tissues (scale bar = 400 μm). **G** NRP1 (green) and EGFR (red) co-localization is examined by the immunofluorescence assay (scale bar = 15 μm) and the nucleus is indicated by DAPI (blue) staining. **H** Exogenous Co-IP assay represents NRP1 could interact with EGFR in 293T cells. **I**, **J** Endogenous Co-IP assay proves NRP1 could interact with EGFR in PC-3 and DU-145 cells. Statistical significance was assessed using a two-tailed *t* test.

GSEA analysis was performed based on the TCGA-PRAD dataset to investigate the potential mechanism of NRP1 in malignant PCa progression. This indicated enrichment of EGFR-related signaling in the samples with highly expressed NRP1 (Fig. 5A, B). Further, RNA stability assay and protein stability assay were performed in PC-3 cells. The results indicated that NRP1 did not lead to EGFR and protein stability (Fig. S3A–C) and the attenuation in phosphorylated EGFR protein led to NRP1 depletion in PC-3 and DU-145 cells (Fig. 5C). Conversely, ectopic overexpression of NRP1 led to an increase in the phosphorylated EGFR protein levels (Fig. 5D). Moreover, it was observed that NRP1 protein was positively correlated with the expression of phosphorylated EGFR protein in PCa tissues (Fig. 5E, F). A previous study reported that NRP1 can act as a co-receptor of multiple tyrosine kinase receptors to promote tumor proliferation and migration [27]. Thus, it can be speculated that NRP1 may interact with EGFR and modulate EGFR phosphorylation. Immunofluorescence assay demonstrated NRP1 co-localized with EGFR in the cell membranes of PC-3 and DU-145 (Fig. 5G). Further, the exogenous Co-IP assay revealed that NRP1 could interact with EGFR in 293T cells (Fig. 5H). Moreover, endogenous Co-IP assay proved NRP1 could interact with EGFR in PC-3 cells (Fig. 5I, J).

**Fig. 6:**
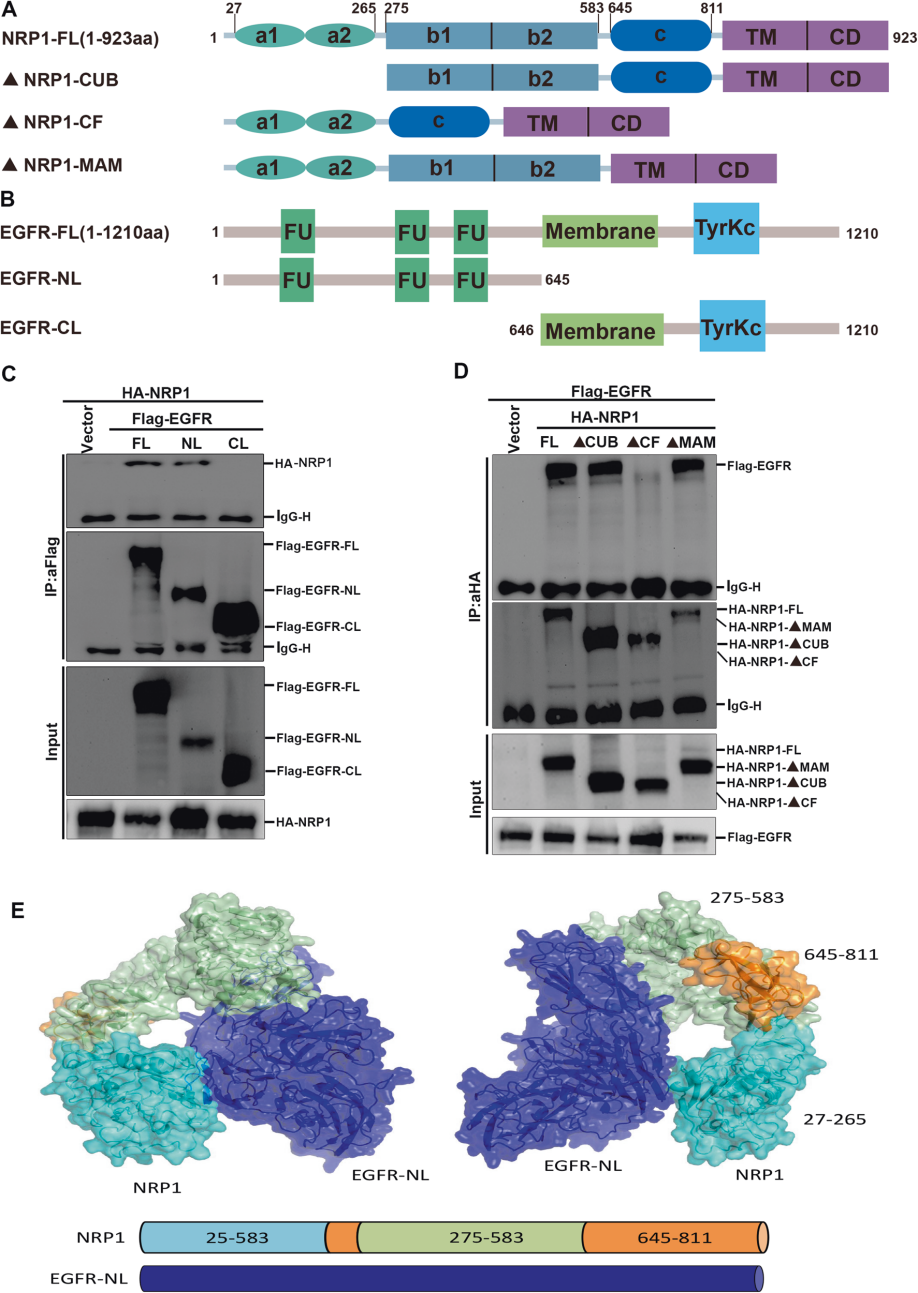
b1/b2 domain of NRP1 interacts with the extracellular domain of EGFR to influence the activation of EGFR. **A**, **B** Schematic diagram of NRP1 and EGFR primary structure and truncated plasmids construction. **C** Identification of the b1/b2 domain of NRP1 mediating its interaction with EGFR. **D** Identification of the extracellular domain of EGFR mediating its interaction with NRP1. **E** Molecular docking analysis shows the interaction domain between NRP1 and EGFR.

The N-terminal region of NRP1 contains three domains, each of which binds to different proteins and mediates different signaling pathways. EGFR contains an extracellular ligand-binding domain at the N-terminus, a transmembrane lipophilic region, and an intracellular region containing the tyrosine kinase domain at the C-terminus. To identify the key region of NRP1 interacting with EGFR, three domain truncation mutants of NRP1 were cloned (Fig. 6A), and EGFR was divided into the extracellular domain EGFR-NL and the intracellular domain EGFR-CL (Fig. 6B). The Co-IP result revealed that NRP1 interacted with the extracellular domain of EGFR (Fig. 6C), and the b1/b2 is the key domain of NRP1 required for this interaction (Fig. 6D). Moreover, the protein molecular docking model also demonstrated that the b1/b2 domain of NRP1 can interact with the extracellular domain of EGFR (Fig. 6E). Thus, these results demonstrated that NRP1 could interact with EGFR, which is mediated by the transmembrane regions of the EGFR and NRP1 b1/b2 domain.

### NRP1 facilitates the oncogenic functions via EGFR/AKT signaling axis

**Fig. 7:**
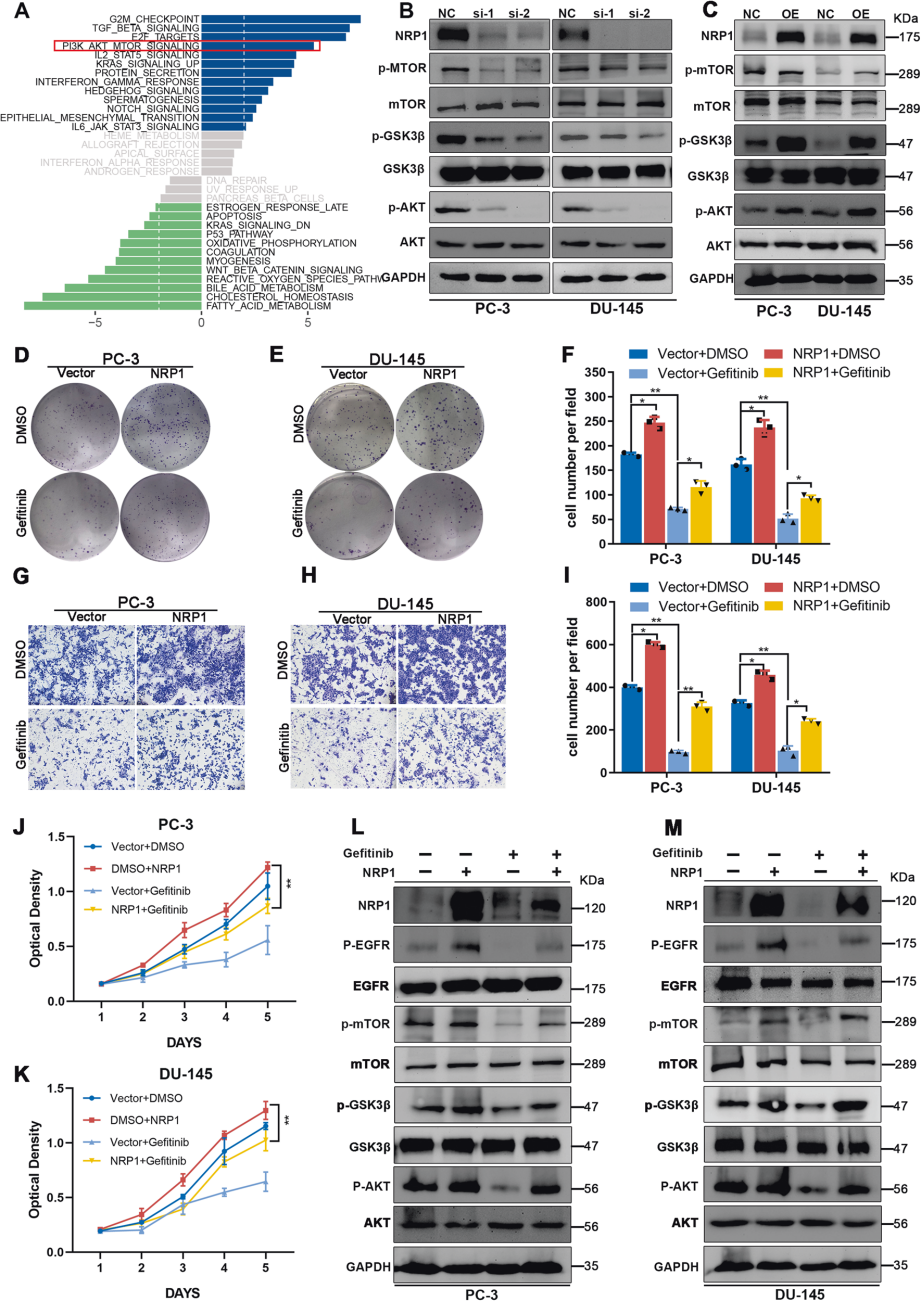
NRP1 promotes PCa cell proliferation through the EGFR/AKT signaling axis. **A** GSVA result indicates the AKT pathway is significantly downregulated in the NRP1-depletion group compared to the control group based on RNA-Seq data of NRP1-depleted DU-145 cells and control cells. **B**, **C** Immunoblot assay elucidates that NRP1 depletion attenuates AKT, GSK3β, and mTOR phosphorylation levels, while ectopic overexpression of NRP1 increases AKT, GSK3β, and mTOR phosphorylation levels. **D**–**F** Clone formation assay and the statistical chart represent EGFR inhibitor gefitinib attenuating clone formation ability induced by NRP1 overexpression in PC-3 and DU-145 cells. **G**–**I** Transwell assay and the statistical chart represent gefitinib attenuates migration ability induced by NRP1 overexpression. **J**, **K** MTT assay elucidates gefitinib attenuates proliferation viability induced by NRP1 overexpression. **L**, **M** Immunoblot results elucidate that gefitinib attenuates AKT, GSK3β, and mTOR phosphorylation levels induced by NRP1 overexpression. Statistical significance was assessed using a two-tailed *t* test. Error bars represent the standard deviations of three independent experiments. *P < 0.01, **P < 0.001.

RNA-Seq analysis was performed using NRP1-depleted DU-145 cells and control cells to investigate the downstream signaling pathways and functional processes that were affected by NRP1-mediated transcriptional regulation. The GSVA result indicated significant downregulation of the AKT pathway in NRP1-depleted DU-145 cells compared to controls (Fig. 7A). The KEGG analysis based on the TCGA-PRAD dataset also indicated enrichment of the AKT pathway in the high NRP1 expression group (Supplementary Fig. S4). Further, the key proteins in AKT pathways were detected after overexpression or knockdown of NRP1. It was observed that the phosphorylation of AKT, GSK3β, and mTOR was downregulated in NRP1-depleted PCa cell lines. Contrarily, ectopic overexpression of NRP1 enhanced the phosphorylation of AKT, GSK3β, and mTOR in PCa cells (Fig. 7B, C). As a previous study reported the AKT pathway to be the downstream signaling pathway of EGFR, it was hypothesized that NRP1 played an oncogenic role through EGFR/AKT signaling axis in PCa. Further, the role of EGFR in NRP1-induced PCa progression was evaluated by using gefitinib, a specific inhibitor of EGFR, in NRP1-overexpressed PCa cells to ascertain the changes in cell proliferation and migration. It was observed that gefitinib obviously retrieval the promotion of proliferation and migration in NRP1- overexpressing PCa cells (Fig. 7D–K). Immunoblot assay demonstrated that PCa cells treated with gefitinib inhibited the NRP1-induced EGFR/AKT signaling axis (Fig. 7L, M). Thus, these results exhibited that NRP1 promoted PCa progression by modulating the activation of the EGFR/AKT signaling pathway.

### EG01377 suppresses PCa progression by inactivating EGFR/AKT signaling axis

**Fig. 8:**
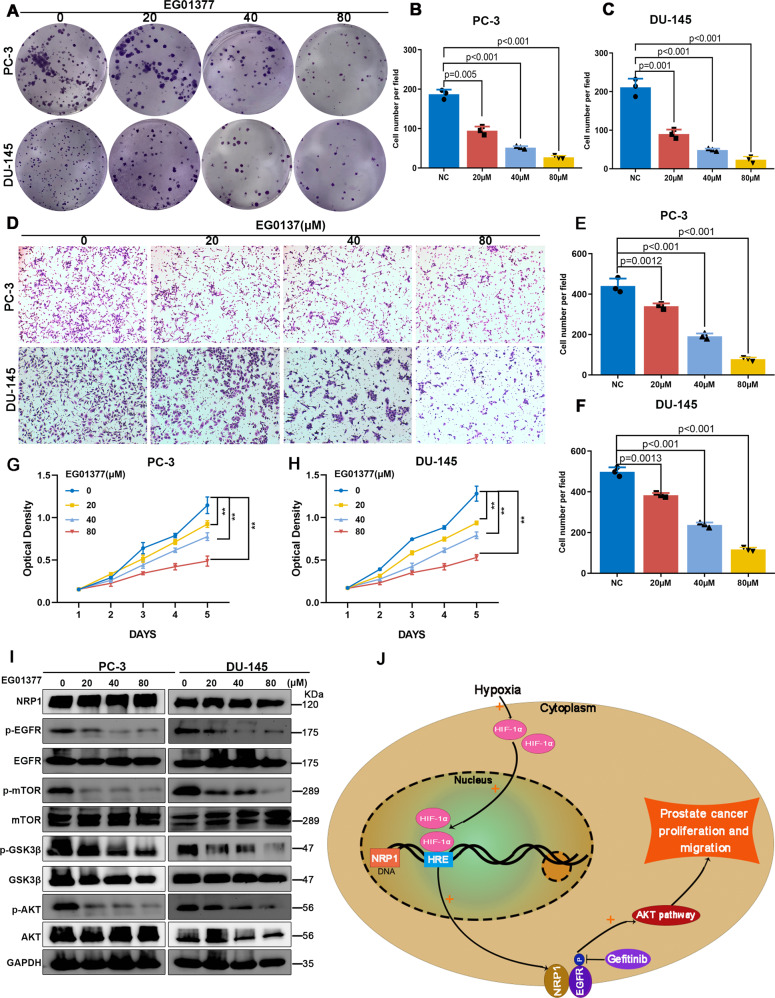
NRP1 inhibitor EG01377 attenuates PCa proliferation and migration via EGFR/AKT signaling axis. **A**–**C** Clone formation assay, **D**–**F** transwell assay, and **G**, **H** MTT assay represent that NRP1 inhibitor EG01377 attenuates clone formation, proliferation, and migration ability in a concentration-dependent manner. **I** Immunoblot results elucidate that EG01377 attenuates AKT, GSK3β, and mTOR phosphorylation levels in a concentration-dependent manner. **J** A schematic model of HIF1α-NRP1-EGFR/AKT signaling axis promoting prostate cancer progression. Statistical significance was assessed using a two-tailed *t* test. Error bars represent the standard deviations of three independent experiments. **P < 0.001.

To investigate the clinical significance of the NRP1 inhibitor, the effects of a novel inhibitor of NRP1 (EG01377) were further evaluated in PCa progression. Different concentrations of EG01377 were used for the treatment of PC-3 and DU-145 cells. MTT and clone formation assay demonstrated that EG01377 significantly attenuated cell proliferation and clone formation ability in a concentration-dependent manner (Figs. 8A–C and 8G, H). The transwell assay also revealed that EG01377 significantly inhibited the migration ability in a concentration-dependent manner (Fig. 8D–F). The immunoblot assay results indicated that EG01377 attenuated the phosphorylation of EGFR, AKT, GSK3β, and mTOR in PC-3 and DU-145 cells (Fig. 8I). Thus, these results indicated that the administration of EG01377 to PCa cells significantly inactivated EGFR/AKT signaling axis, thereby suppressing PCa progression.

## Discussion

PCa, one of the most common urinary tumors, is the fifth leading cause of cancer-related deaths in men and has become a global threat to men’s health. However, the molecular mechanisms underlying PCa progression are still unknown, leading to poor prevention and treatment strategies for advanced PCa, especially CRPC and mCRPC. Therefore, a comprehensive study of molecular biological mechanisms and cellular pathways underlying PCa progression can help to explore novel intervention strategies and drug targets for PCa.

In this study, the focus was on NRP1 and its oncogenic role in PCa. It was observed that NRP1 was overexpressed in PCa, positively associated with adverse clinicopathologic factors, and an independent predictor for poor survival. In PCa cells, NRP1 promoted malignant phenotypes, including cell viability and migration, tumor growth, and metastasis. Mechanistically, NRP1 binding triggered EGFR phosphorylation, thereby activating the AKT/mTOR pathway to promote malignant PCa cell behavior. Moreover, HIF1α is involved in the transcriptional regulation of NRP1, as shown in Fig. 8J. It was verified that EG10377, a specific inhibitor of NRP1 attenuated PCa progression, and demonstrated that targeting NRP1 with a genetic or small-molecule approach significantly inhibited PCa tumor growth.

NRP1 is a single-pass transmembrane glycoprotein belonging to the neuropilin family. A previous study reported that NRP1 is highly expressed in several tumors, and an increase in its level correlated with tumor progression [28–30], thus highlighting the crucial role played by NRP1 in the occurrence and development of cancer. NRP1 acts as an oncogene and is involved in the progression of cancer, including breast cancer [15], lung cancer [31], bladder cancer [32], and renal cell carcinoma [33]. However, there is a lack of research on NRP1 in PCa, and the underlying mechanism is not clear. In this study, bioinformatics analysis was used to indicate that NRP1 was significantly highly expressed in PCa, and may be involved in the occurrence and progression of PCa. Prognostic analysis revealed that NRP1 could be an independent risk factor for PCa prognosis. Further, it was demonstrated that NRP1 depletion in PCa cells could attenuate the proliferation of PCa cells by inducing G1 phase cell cycle arrest. At the same time, it was observed that NRP1 depletion can reverse the EMT state of PCa cells and inhibit their migration. Thus, these results indicated that NRP1 is an important molecule in the regulation of PCa progression.

Hypoxia is a common feature of solid malignancies including PCa [34]. Under hypoxic conditions, the hypoxia-inducible factor 1 (HIF1) pathway is activated and generally induces the expression of downstream oncogenes in tumor cells. HIF1α is an important transcription factor of the HIF1 pathway, which is associated with hypoxic stress [35]. Mounting evidence has suggested that overexpression of HIF1α in PCa activated several signaling pathways involved in cellular differentiation, metastasis, tumor progression, angiogenesis, and resistance to castration or chemotherapy [36–39]. Previous research indicated that NRP1 was correlated with HIF1α expression in pancreatic cancer and non-small cell lung cancer (NSCLC) [31, 40]. In this study, NRP1 was shown to have a significant positive correlation with HIF1α in PCa. Furthermore, HIF1α accumulation led to transcriptional activation of NRP1 expression. Dual-luciferase assay demonstrated that NRP1 was a direct transcriptional target of HIF1α. An increase in NRP1 expression by HIF1α in PCa may be one of the critical mechanisms of PCa progression.

Although high expression of NRP1 is caused by HIF1α, the exact mechanism involved in the promotion of cell growth in PCa by NRP1 needs to be investigated. In this study, GSEA revealed that NRP1 regulated EGF/EGFR signaling axis in PCa. EGFR is a member of the ErbB transmembrane growth factor receptor family [41]. It is primarily composed of an extracellular ligand-binding region, a transmembrane hydrophobic region, and an intracellular tyrosine kinase active region. The EGFR ligands are epidermal growth factor, transforming growth factor alpha, etc. The binding of EGFR ligands induces the formation of EGFR homodimers or heterodimers, which subsequently trigger intracellular tyrosine kinases and initiate autophosphorylation processes. The activation of EGFR has been widely proven to promote cancer progression, metastasis, recurrence, and drug resistance [42, 43]. The results from this study proved that NRP1 induced the phosphorylation of EGFR by direct NRP1-EGFR interactions. A series of biochemical assays demonstrated that the b1/b2 domain of NRP1 interacted with the extracellular domain of EGFR to regulate the phosphorylation of EGFR.

Aberrant EGFR can modulate several downstream pro-oncogenic signaling pathways, including PI3K/AKT pathway, Ras/Raf/MAPK pathway, and JAK/STAT pathway [44–46]. These pathways are closely associated with the proliferation, invasiveness, and angiogenesis of cancer, ultimately leading to the malignant biological behavior in cancer [47–49]. KEGG signaling pathway analysis demonstrated that NRP1 was closely related to the AKT pathway. Immunoblot results demonstrated that NRP1 depletion attenuated the phosphorylation of AKT, GSK3β, and mTOR proteins. Several studies have demonstrated that the AKT-mTOR pathway promoted PCa progression and mediated CRPC state [50–52]. Therefore, NRP1 may play a biological role in PCa by activating the downstream AKT-mTOR pathway.

In conclusion, this study demonstrated a novel signaling regulatory axis in PCa progression. As shown in Fig. 8J, in the hypoxic tumor microenvironment, elevated NRP1 expression was mediated by transcriptional regulation of HIF1α. Moreover, NRP1 can activate EGFR phosphorylation, subsequently activating the downstream AKT-mTOR pathway, and ultimately contributing to tumor progression in PCa. This study indicated that NRP1 may serve as a potential predictor and therapeutic target in PCa progression.

## Supplementary information

Original Data File

Supplementary Figures

Table S1

reproducibility checklist

## Data Availability

Data will be made available on reasonable request.
