# Supplementary figures and images for: NRP1 promotes prostate cancer progression via modulating EGFR-dependent AKT pathway activation
Source: Cell Death Dis. 2023 Feb 25;14(2):159. doi: 10.1038/s41419-023-05696-1 (PMC9958327; doi:10.1038/s41419-023-05696-1)

**Figure 2**

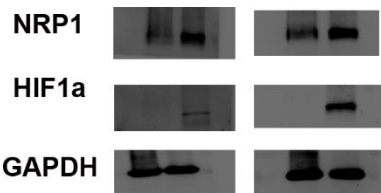

**Figure 3**

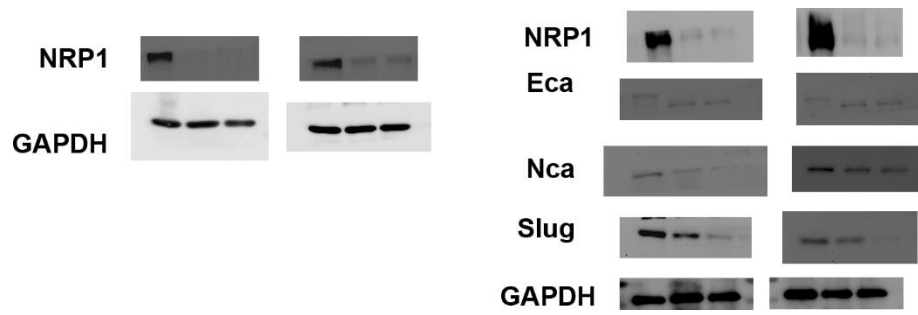

**Figure 4**

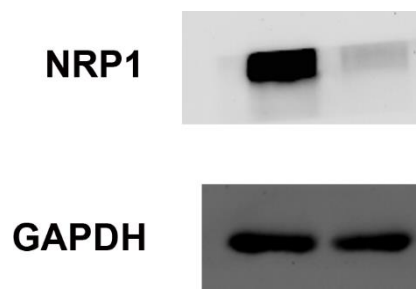

**Figure 5**

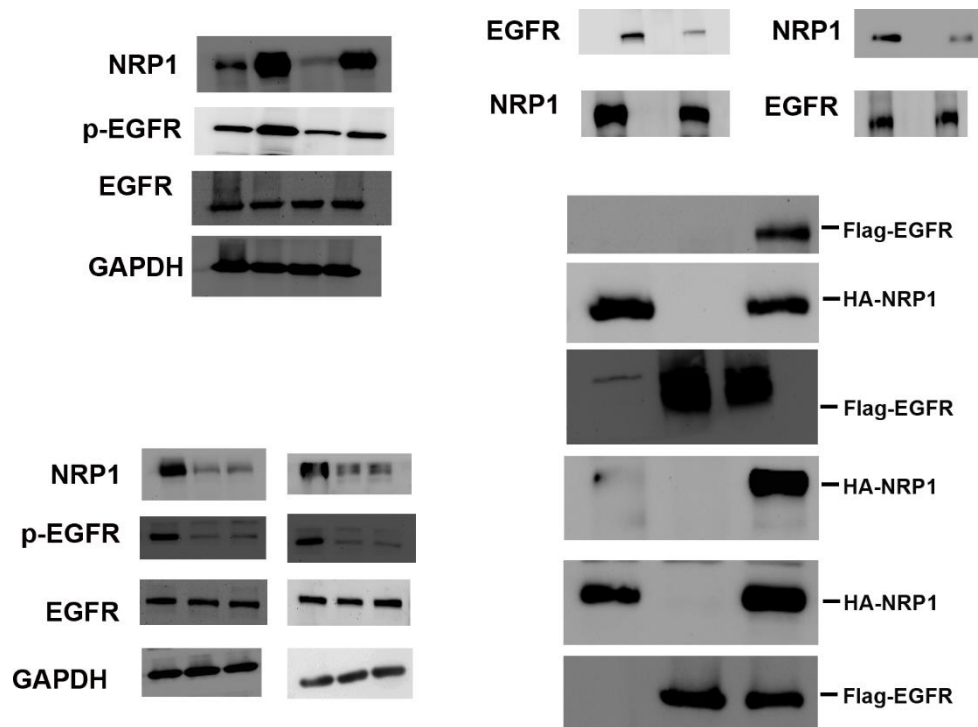

**Figure 6**

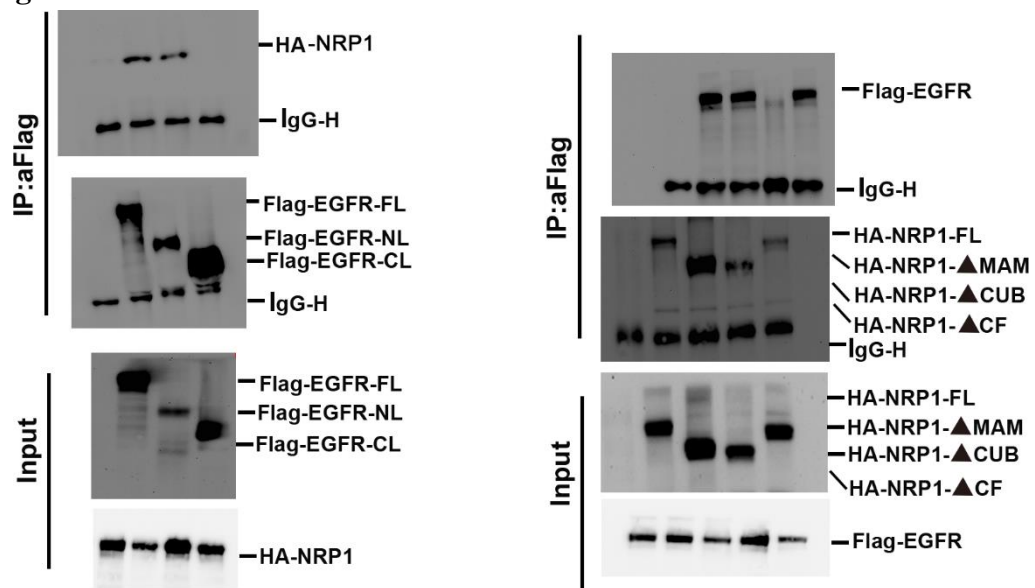

Figure 7

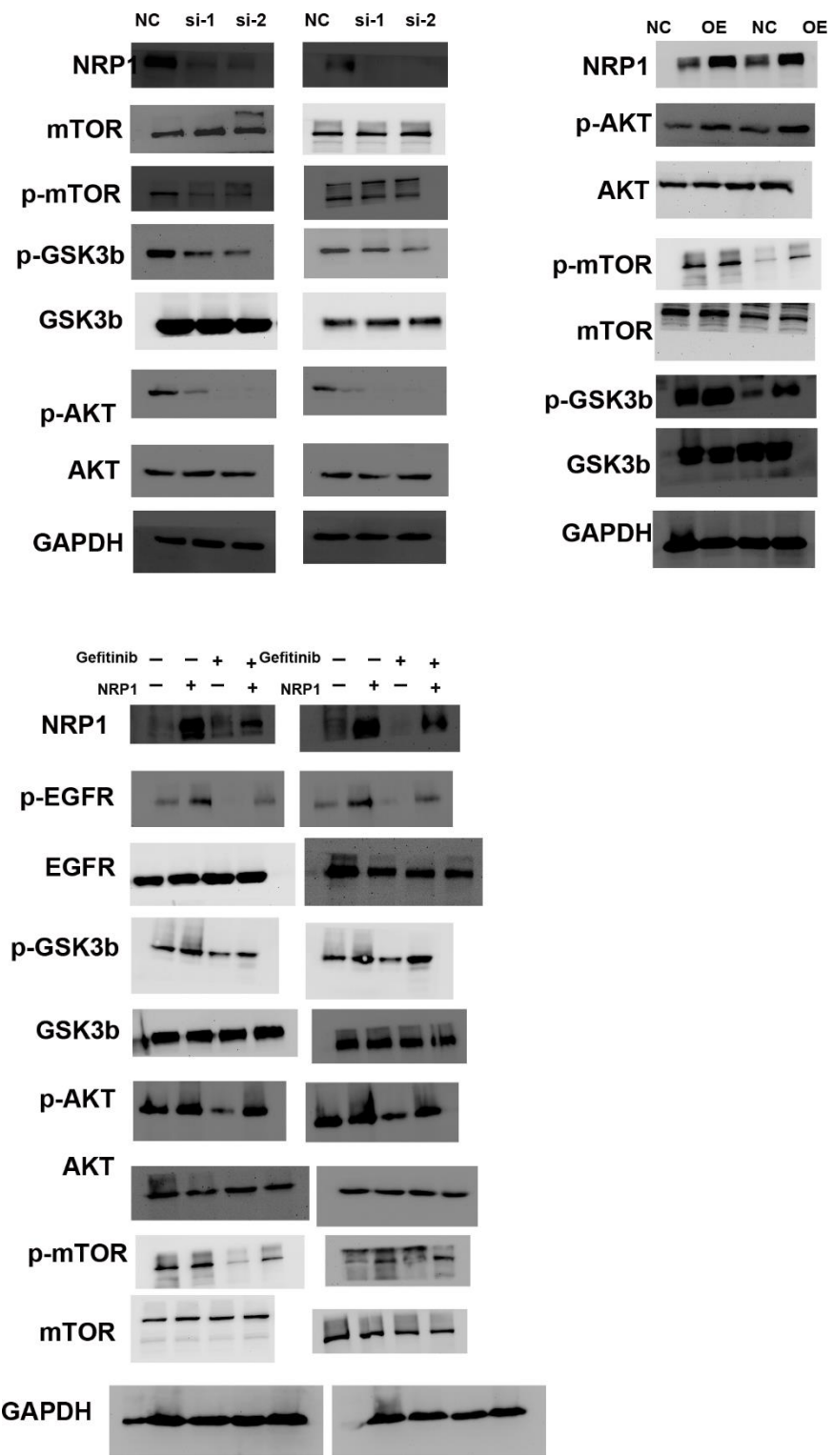

**Figure 8**

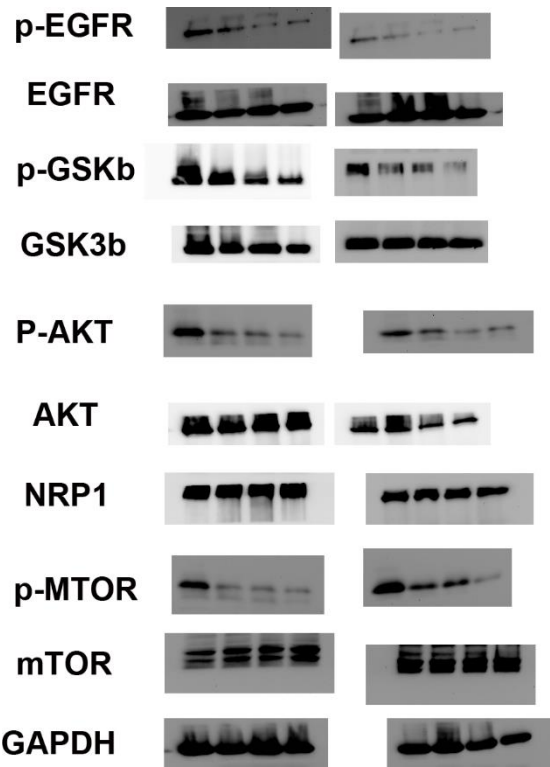

**Figure S1**

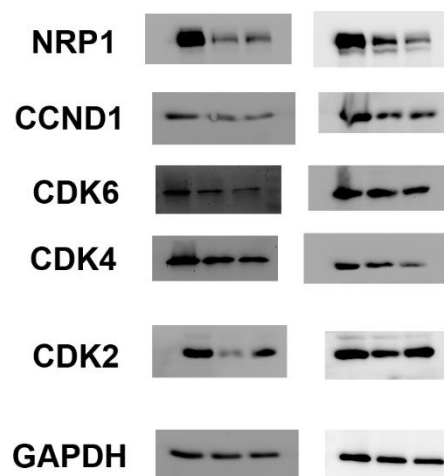

Figure S2

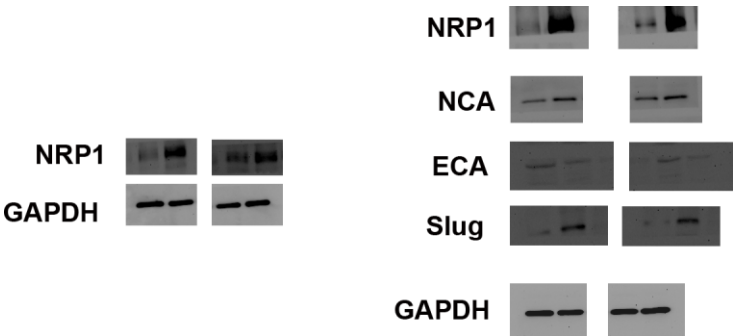

**Figure S3**

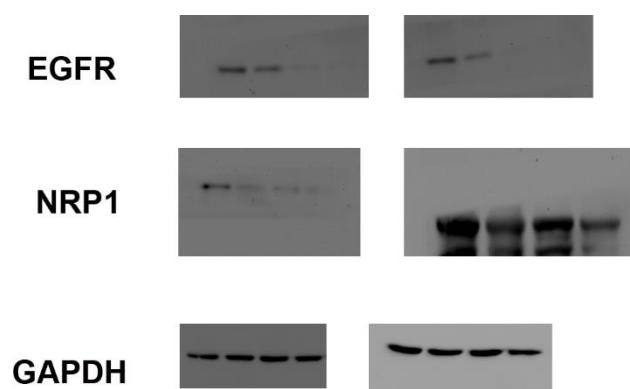

Supplement: Supplementary file 1 — Original Data File [file 41419_2023_5696_MOESM1_ESM.pdf]
