# Supplementary material for: NRP1 promotes prostate cancer progression via modulating EGFR-dependent AKT pathway activation
Source: Cell Death Dis. 2023 Feb 25;14(2):159. doi: 10.1038/s41419-023-05696-1 (PMC9958327; doi:10.1038/s41419-023-05696-1)
Supplement: Supplementary file 2 — Supplementary Figures [file 41419_2023_5696_MOESM2_ESM.docx]

**
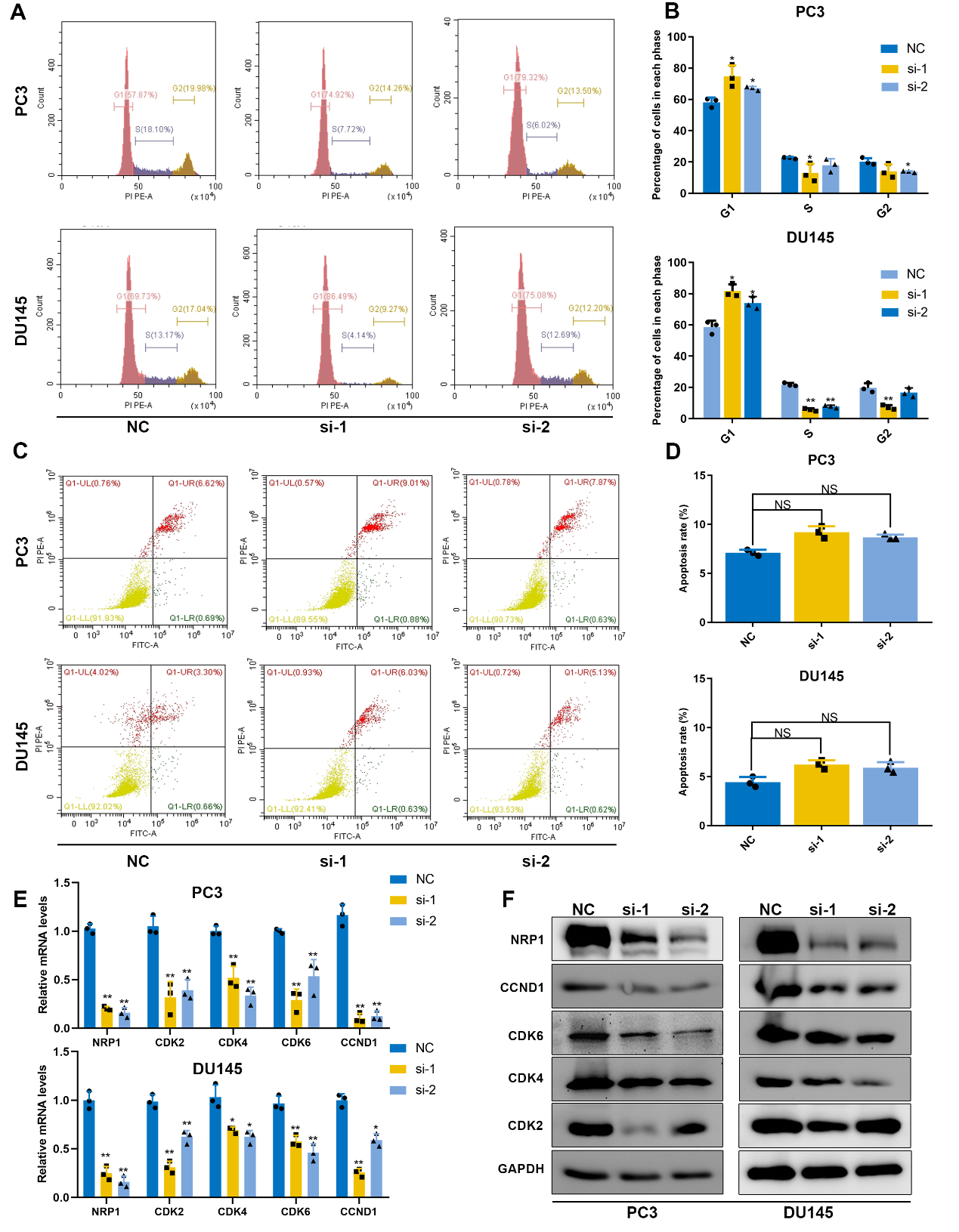
Supplementary Figures**

**Figure S1. NRP1 regulates the G1-S phase transition and alters the expression of cell-cycle regulators in PCa cells.** (**A-B**) Flow cytometry represented the alteration of cell cycle after depletion of NRP1 in PC-3 and DU-145 cells. (**C-D**) Flow cytometry represented the alteration of cell apoptosis after depletion of NRP1 in PC-3 and DU-145 cells. (**E**) qRT-PCR assay represnts the alteration of cell-cycle related genes in PC-3 and DU-145 cells after NRP1 depletion. (**F**) Immunoblot assay shows the alteration of cell-cycle related proteins in PC-3 and DU-145 cells after NRP1 depletion. Statistical significance was assessed using two-tailed t-tests. *p<0.01, **p<0.001.


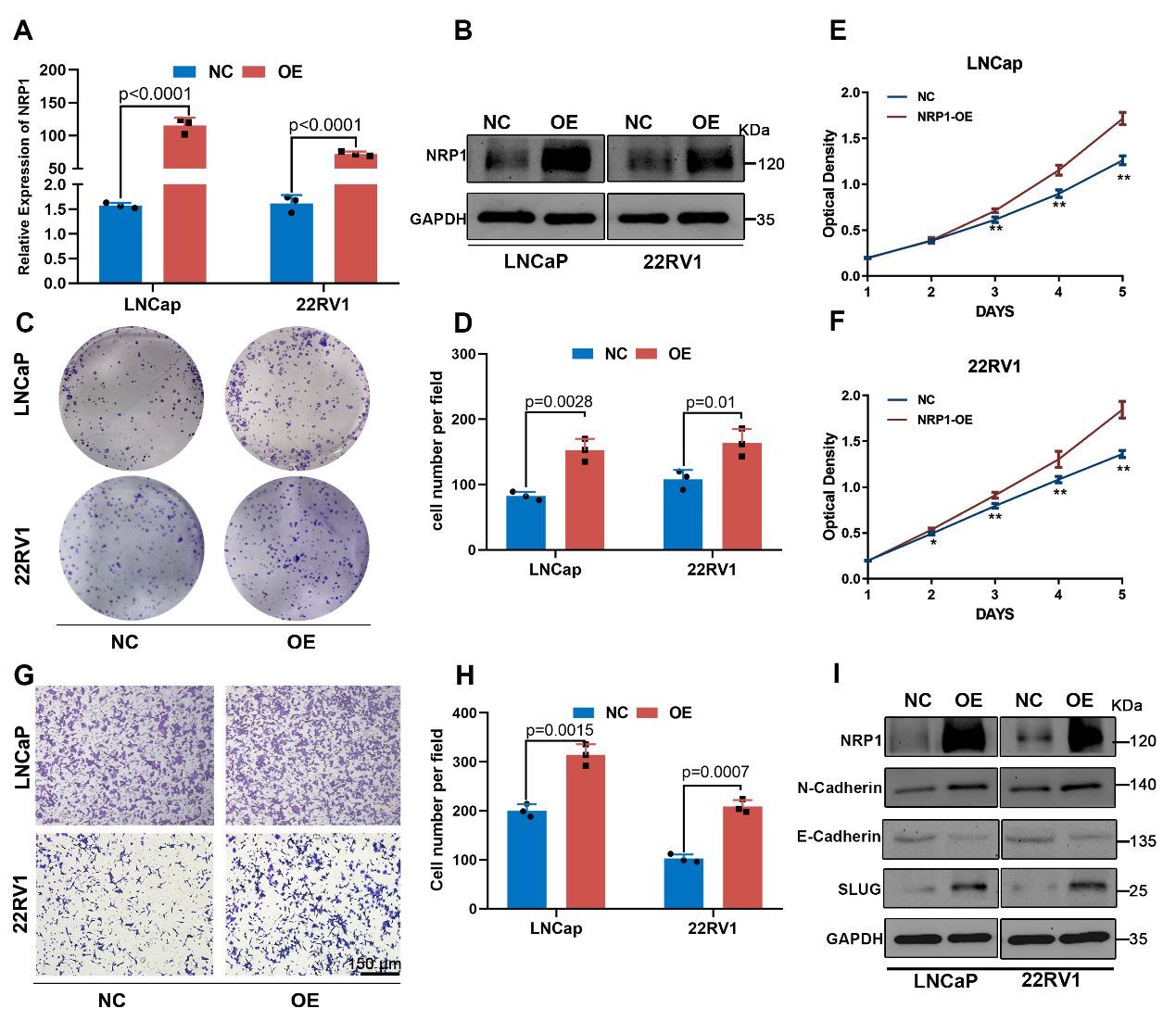
**Figure S2. NRP1 overexpression promotes PCa cells proliferation and migration in vitro.** (**A-B**) qRT-PCR and immunoblot assays valuate transfection efficiency of NRP1 overexpression plasmid in LNCap and 22RV1 cells (**C-D**) Clone formation assay and the statistical chart represent NRP1 overexpression increases clone formation viability in LNCap and 22RV1 cells. (**E-F**) MTT assays reveals that NRP1 overexpression increases cell viability in LNCap and 22RV1 cells. (**G-H**) Transwell assay and the statistical chart demonstrated NRP1 overexpression increases cell migration in LNCap and 22RV1 cells. (**I**) Immunoblot assay shows EMT-related proteins in LNCap and 22RV1 cells after NRP1 ectopic overexpression. Statistical significance was assessed using two-tailed t-tests. *p<0.01, **p<0.001.

**
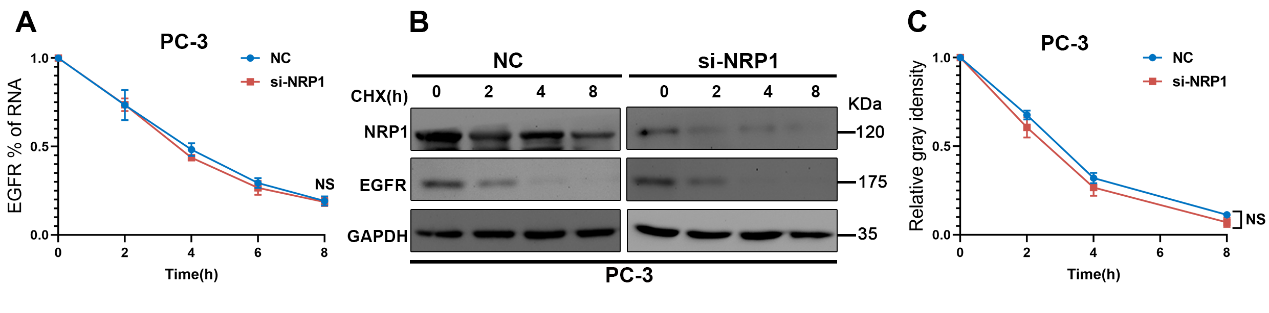
**

**Figure S3. NRP1 don’t modulate RNA stability and protein stability of EGFR.** (**A**) RNA stability assay represents that the RNA stability of EGFR has no alternation after NRP1 depletion. (**B-C**) Protein stability assay and the statistical chart represent that the protein stability of EGFR has no alternation after NRP1 depletion. NS, not significant.


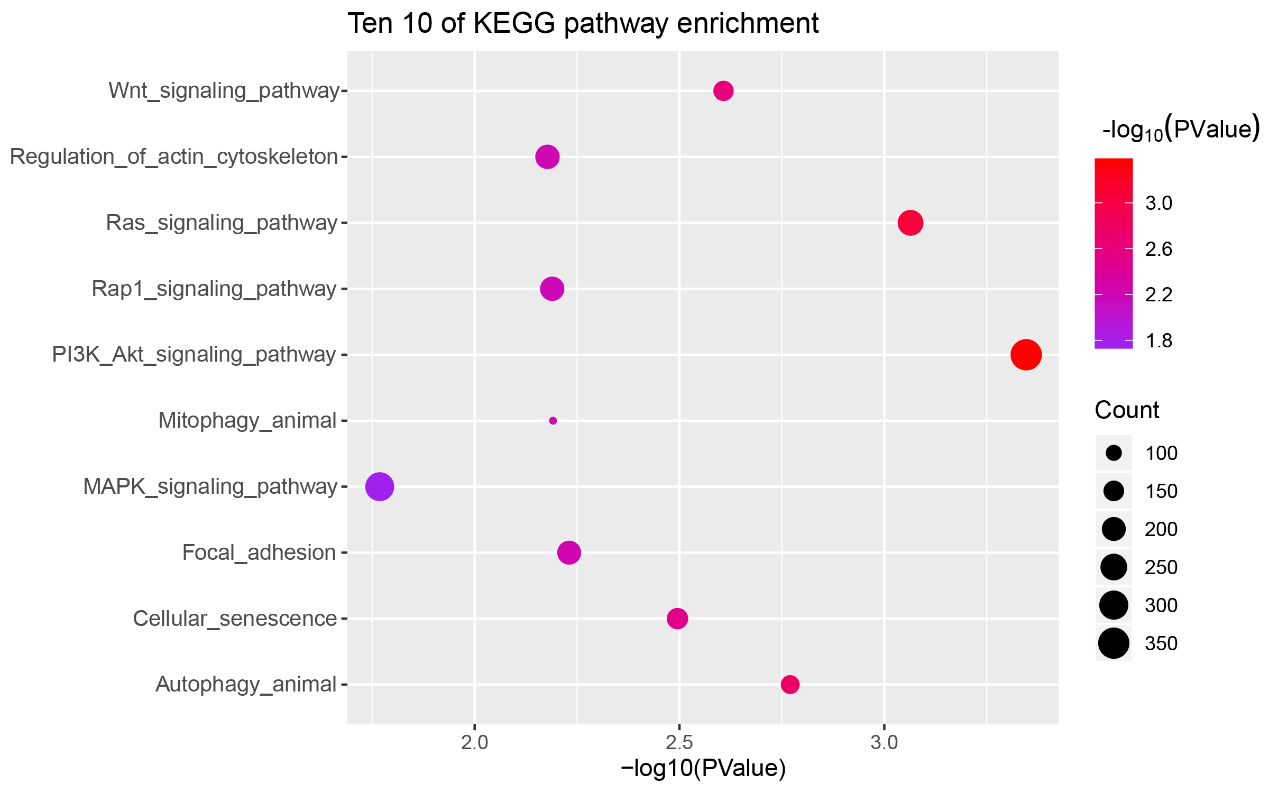
**Figure S4.** **KEGG pathway analysis based on NRP1 expression in TCGA-PRAD dataset.** the result showed the top 10 of KEGG pathway enrichment, and AKT pathway was enriched in NRP1 highly expressed group.

**Table S1 The specific primer sequences**

| **ID** | **Primer** | Primer sequences **(5'to3')** | **Base count** |
| --- | --- | --- | --- |
| 1 | NRP1-F | GGCGCTTTTCGCAACGATAAA | 21 |
| 2 | NRP1-R | TCGCATTTTTCACTTGGGTGAT | 22 |
| 3 | CDK2-F | CCAGGAGTTACTTCTATGCCTGA | 23 |
| 4 | CDK2-R | TTCATCCAGGGGAGGTACAAC | 21 |
| 5 | CDK4-F | ATGGCTACCTCTCGATATGAGC | 22 |
| 6 | CDK4-R | CATTGGGGACTCTCACACTCT | 21 |
| 7 | CDK6-F | GCTGACCAGCAGTACGAATG | 20 |
| 8 | CDK6-R | GCACACATCAAACAACCTGACC | 22 |
| 9 | CCND1-F | GCTGCGAAGTGGAAACCATC | 20 |
| 10 | CCND1-R | CCTCCTTCTGCACACATTTGAA | 22 |
| 11 | EGFR-F | AGGCACGAGTAACAAGCTCAC | 21 |
| 12 | EGFR-R | ATGAGGACATAACCAGCCACC | 21 |
| 13 | HIF1α-F | GAACGTCGAAAAGAAAAGTCTCG | 23 |
| 14 | HIF1α-R | CCTTATCAAGATGCGAACTCACA | 23 |
| 15 | GAPDH-F | GGAGCGAGATCCCTCCAAAAT | 21 |
| 16 | GAPDH-R | GGCTGTTGTCATACTTCTCATGG | 23 |
