# Supplementary material for: NRP1 promotes prostate cancer progression via modulating EGFR-dependent AKT pathway activation
Source: Cell Death Dis. 2023 Feb 25;14(2):159. doi: 10.1038/s41419-023-05696-1 (PMC9958327; doi:10.1038/s41419-023-05696-1)
Supplement: Supplementary file 3 — Table S1 [file 41419_2023_5696_MOESM3_ESM.docx]

**Table S1 The specific primer sequences**

| **ID** | **Primer** | Primer sequences **(5'to3')** | **Base count** |
| --- | --- | --- | --- |
| 1 | NRP1-F | GGCGCTTTTCGCAACGATAAA | 21 |
| 2 | NRP1-R | TCGCATTTTTCACTTGGGTGAT | 22 |
| 3 | CDK2-F | CCAGGAGTTACTTCTATGCCTGA | 23 |
| 4 | CDK2-R | TTCATCCAGGGGAGGTACAAC | 21 |
| 5 | CDK4-F | ATGGCTACCTCTCGATATGAGC | 22 |
| 6 | CDK4-R | CATTGGGGACTCTCACACTCT | 21 |
| 7 | CDK6-F | GCTGACCAGCAGTACGAATG | 20 |
| 8 | CDK6-R | GCACACATCAAACAACCTGACC | 22 |
| 9 | CCND1-F | GCTGCGAAGTGGAAACCATC | 20 |
| 10 | CCND1-R | CCTCCTTCTGCACACATTTGAA | 22 |
| 11 | EGFR-F | AGGCACGAGTAACAAGCTCAC | 21 |
| 12 | EGFR-R | ATGAGGACATAACCAGCCACC | 21 |
| 13 | HIF1α-F | GAACGTCGAAAAGAAAAGTCTCG | 23 |
| 14 | HIF1α-R | CCTTATCAAGATGCGAACTCACA | 23 |
| 15 | GAPDH-F | GGAGCGAGATCCCTCCAAAAT | 21 |
| 16 | GAPDH-R | GGCTGTTGTCATACTTCTCATGG | 23 |
